# Subtype-Specific HIV-1 Protease and the Role of Hinge and Flap Dynamics in Drug Resistance: A Subtype C Narrative

**DOI:** 10.3390/v17081044

**Published:** 2025-07-26

**Authors:** Dean Sherry, Zaahida Sheik Ismail, Tshele Mokhantso, Yasien Sayed

**Affiliations:** Protein Structure-Function Research Laboratory, University of the Witwatersrand, Johannesburg 2000, South Africa

**Keywords:** HIV-1 subtype C protease, hinge region dynamics, flap flexibility, genetic diversity, drug resistance mutations, protease inhibitors, RNA aptamers, allosteric inhibitors, dual-action inhibitors, peptide inhibitors

## Abstract

The HIV-1 aspartic protease is an effective target for the treatment of HIV/AIDS. Current therapy utilizes a selection of nine protease inhibitors (PIs) in combination with other classes of antiretroviral drugs. Although PIs were originally developed based on the knowledge of the HIV-1 subtype B protease, the existence of other HIV-1 subtypes and the effects of drug resistance on currently available PIs have become a major challenge in the treatment of HIV/AIDS. Specifically, the HIV-1 subtype C accounts for more than half of the global HIV infections. Considering the importance and relevance of the subtype C virus, in this timely review we discuss the effect of polymorphisms in the HIV-1 subtype C protease on drug resistance, flap flexibility, and hinge region dynamics. We discuss novel paradigms of protease inhibition that attempt to overcome the limitations of currently available inhibitors which fall short considering genetic diversity and resistance mutations.

## 1. Introduction

The Human Immunodeficiency Virus (HIV), the causative agent of Acquired Immunodeficiency Syndrome (AIDS), continues to impose a significant global socio-economic impact, with developing countries generally being most greatly affected. The latest statistics on global infections estimate that ~39 million people were living with HIV as of 2023 [1]. The HIV/AIDS epidemic is particularly prominent in sub-Saharan Africa where roughly 70% of global infections occur: a fact that is reflected in these countries exhibiting the highest morbidity and mortality rates [1].

**Figure 1:**
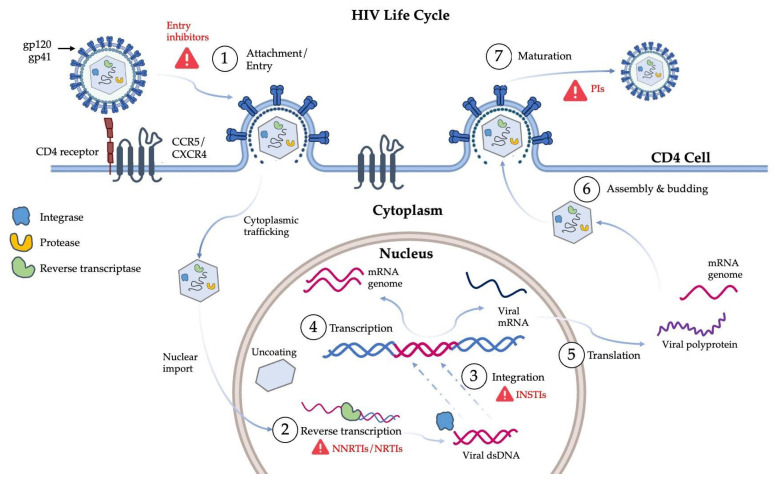
The life cycle of HIV. The essential stages of the life cycle are numbered and correspond to: (1) attachment and viral entry via CD4 and co-receptors; (2) reverse transcription; (3) integration; (4) transcription; (5) translation; (6) assembly and budding; (7) maturation. Red triangles indicate the major drug targets at specific stages [2,3]. Image generated using Microsoft PowerPoint.

Since the discovery of HIV as the causative agent of AIDS, a significant amount of research has focused on understanding the biomolecular interactions that govern the life cycle of the virus with the goal of identifying promising drug targets. The life cycle of HIV (Figure 1) begins with attachment of the viral spike glycoproteins (gp120/gp41) to the CD4 receptor present on the cell membrane of host CD4+ T-lymphocytes. This interaction is stabilized by binding to CCR5 or CXCR4 co-receptors, enabling fusion of the viral envelope with the host cell membrane. Following entry, the viral core containing the single-stranded, positive-sense RNA genome and associated enzymes is released into the cytoplasm. Contrary to earlier models, it is now well established that reverse transcription does not occur freely in the cytoplasm but rather within the intact or partially disassembled viral capsid. The viral reverse transcriptase (RT) synthesizes a complementary negative-sense DNA strand using the RNA genome as a template [2], followed by synthesis of a complementary, positive-sense DNA strand, forming a double-stranded DNA (dsDNA) molecule. This process is thought to be largely completed either within the capsid core as it traffics through the cytoplasm or upon entry into the nucleus. The resulting pre-integration complex, which includes the dsDNA and the viral integrase (IN) enzyme, facilitates integration of the viral genome into the host cell’s chromosomal DNA [2]. The integrated viral genome is translated into viral polyprotein precursors, namely, Gag and Gag-Pol. These polyproteins, along with several copies of the ssRNA viral genome, are transported to the cell membrane. Once the necessary viral components are assembled, membrane envelopment is initiated, followed by budding, resulting in the production of an immature virion [3]. Finally, maturation of the virion occurs through the action of the viral protease (PR), which cleaves the Gag and Gag-Pol polyproteins into enzymes and structural proteins. The resultant virion is mature and capable of infecting subsequent host cells.

The identification of key steps in the virus life cycle has enabled the development of several classes of antiretroviral drugs targeting crucial steps in the production of infectious virions (Figure 1). Highly active antiretroviral therapy (HAART) makes use of up to twenty different drug molecules including nucleoside reverse transcriptase inhibitors (NRTIs), non-nucleoside reverse transcriptase inhibitors (NNRTIs), protease inhibitors (PIs), integrase strand transfer inhibitors (INSTIs), and fusion/entry inhibitors (Figure 1).

### 1.1. Genetic Diversity of HIV-1

The HIV-1 genome has three major genes typical of the *Retroviridae* family, namely, Group-specific antigen (Gag), Polyprotein (Pol), and Envelope (Env). Gag encodes the viral structural proteins such as the Matrix (MA), Capsid (CA), and Nucleocapsid (NC), which account for approximately half of the mass of the HIV-1 virion. Pol encodes the enzymatic proteins responsible for the viral replication process, namely, the RT, IN and PR enzymes. The HIV-1 env gene encodes the envelope glycoprotein precursor gp160, which is synthesized in the endoplasmic reticulum and later cleaved by host furin-like proteases in the Golgi apparatus. The cleavage produces the following two non-covalently associated subunits: gp120, which binds to CD4 and a co-receptor (CCR5 or CXCR4) on the target cell, and gp41, which anchors the glycoprotein to the viral membrane and drives the fusion process necessary for viral entry [4]. The HIV-1 genome additionally has the regulatory genes Tat and Ref and the accessory genes Vif, Vpr, Vpu, and Nef [5]. One of the key characteristics of HIV-1 is its high genetic diversity [6]. Three principal mechanisms contribute significantly to the genetic diversity observed within the virus. Firstly, the rapid replication rate of HIV, estimated to produce approximately 10^10^ virions daily, serves as a substantial driver of genetic variation [7]. Secondly, viral recombination plays a crucial role by facilitating the reassortment of genetic mutations across different viral genomes. Lastly, the inherent error-prone replication process of the RT enzyme, characterized by its low fidelity, further contributes to the accumulation of genetic diversity within HIV [6]. In fact, during replication, the error-prone nature of RT introduces up to 1 error for every 2000 base incorporations [8].

**Figure 2:**
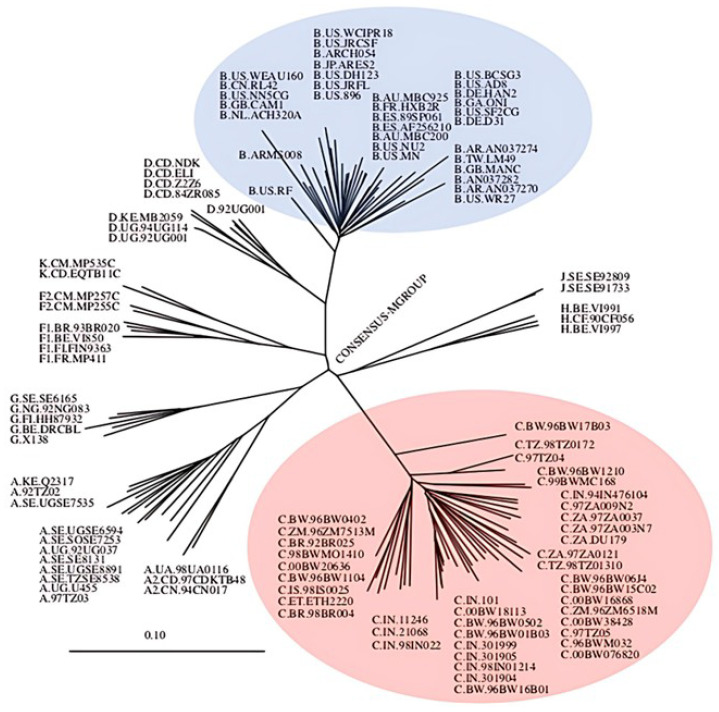
Radial phylogenetic tree of HIV-1 subtypes within the consensus M group. Each sequence is labeled with the subtype (indicated by a letter), country of origin, and a unique identity code. Subtype B and subtype C sequences are highlighted in blue and red, respectively. The tree was constructed using data from the Los Alamos HIV Sequence Database www.hiv.lanl.gov (accessed on 5 May 2025).

To date, two types of HIV have been identified: type 1 (HIV-1) and type 2 (HIV-2). HIV-1 has been shown to be more virulent than HIV-2 and accounts for the majority of global infections [9]. As such, global research efforts have focused on HIV-1. HIV-1 is further classified into the following groups: Group M (main), N (non-M or non-O), O (outlier), and P (a group given its own name due to how different it is from M, N and O groups) [10]. The group which is predominantly responsible for the global HIV/AIDS pandemic is group M. This group is further classified in terms of subtypes, namely, subtype A, B, C, D, F, G, H, J, and K, and circular recombinant forms (CRFs), as illustrated in Figure 2. CRFs are epidemic strains that arise from the recombination of various strains of HIV [10]. Among all the subtypes within group M, subtype B is the most extensively studied and is predominant in the infections found in developed regions such as North America and Western Europe. These regions, often referred to as first-world countries, possess the necessary infrastructure, financial resources, and technological capacity to conduct advanced HIV research. Consequently, the majority of scientific studies in these countries have concentrated on HIV-1 subtype B. This focus has led to the development of drug treatments primarily targeted against subtype B, despite it accounting for only 12% of global HIV infections. In contrast, the HIV-1 subtype C which accounts for more than 50% of global infections has remained largely understudied [11,12,13]. A greater genetic variation exists within HIV-1 subtype C (Figure 2; red) compared to all other HIV-1 subtypes including subtype B (Figure 2; blue). Although the subtype B-derived therapeutic interventions have successfully decreased fatalities and the spread of HIV-1, there has been an increase in the number of non-B subtype infections globally. These variants may evade current therapies, leading to increased drug resistance and treatment failures. Of particular concern is HIV-1 subtype C, which poses significant challenges in treatment efficacy and control.

### 1.2. The Global Predominating Subtype: HIV-1 Subtype C

As mentioned previously, the HIV-1 subtype C is the predominant subtype in developing countries including Brazil, China, India, and South Africa. The South African HIV-1 subtype C protease (C-SA PR) is, in essence, the consensus subtype C PR (Los Alamos HIV Sequence Database, (www.hiv.lanl.gov/). The subtype C accounts for approximately 70% of infections in sub-Saharan Africa. There is evidence to suggest that the efficacy of inhibitors against subtype C is less than that of subtype B [11,12,14,15]. It has been postulated that the discrepancy in the efficacy of antiretroviral therapy across subtypes is due to quantifiable differences in the structural and functional properties of the viral protein targets. All currently available FDA-approved antiretrovirals were designed using subtype B viral proteins as templates for rational drug design. However, as the scope of HIV research has increased to include HIV-1 non-B subtypes, it has become increasingly clear that certain crucial differences exist between antiretroviral drug targets across subtypes. In particular, the PR exhibits significantly decreased drug susceptibility in HIV-1 subtype C [11,12,14,15], perhaps suggesting that current antiretroviral drugs lack broad-spectrum efficacy across different HIV-1 subtypes. Notably, the subtype C PR has been shown to be structurally and dynamically different from the subtype B PR, which may explain the observed differences in drug efficacy (this is explored further in the subsection Naturally Occurring Polymorphisms in the HIV Protease).

Gag is the main structural polyprotein in HIV and its cleavage, by PR, results in the production of mature HIV virions [16,17,18]. During HIV maturation, the scissile bonds on the Gag polyprotein are targeted and cleaved by the PR, a process that is crucial for viral development. Therefore, these cleavage sites have been simulated in efforts to design competitive PIs which are capable of prohibiting viral maturation [19]. However, Gag mutations have previously been shown to alter drug susceptibility, particularly when they have coevolved with mutations in the PR [20,21,22]. The cleavage sites on HIV-1 subtype C Gag polyproteins p17/p24, p2/NC, and NC/p1 were shown to have the highest genetic variation in comparison to other Group M subtypes. Such variability in the Gag cleavage sites between subtypes may immensely affect the pathogenesis, viral fitness, and drug-response of HIV [19,23,24].

The Gag polyprotein plays a significant role in the replicative capacity of HIV. Notably, a comparison of the Gag-PR-mediated replication capacity of HIV-1 subtype B and C revealed a reduced replicative capacity in subtype C [25]. Similar studies have also suggested that subtype C has a reduced viral fitness compared to other subtypes within Group M [26,27]. A decrease in replication capacity has been attributed to the presence of residues L483 and Y484 in the subtype B Gag sequence, which are absent in the subtype C Gag. Deletion of these residues in the subtype B Gag consistently resulted in a decreased viral replication capacity in comparison to when these residues are present [25]. This natural difference in subtype C may facilitate a slower progression of subtype C infections and, thus, increase the opportunity for new transmission events. Some studies suggest that the reduced replication kinetics of HIV-1 subtype C may increase the half-life of productively infected cells and these infected cells, if transmitted in genital fluid, may increase transmission risk compared to free virions [25].

### 1.3. Structure of the HIV-1 Subtype C Protease

**Figure 3:**
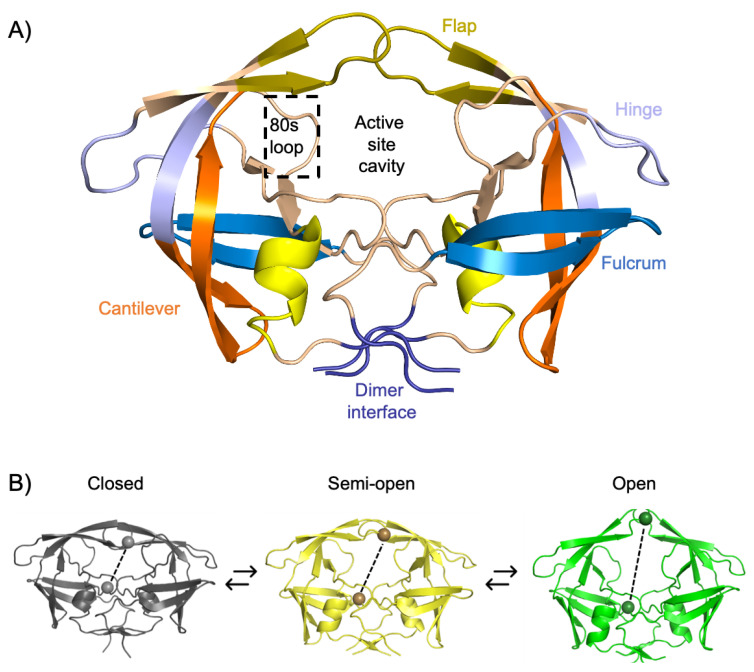
Structural features and flap conformations of HIV-1 C-SA PR. (**A**) Key structural regions are color-coded: dimer interface (purple; residues 1–4 and 94–99), fulcrum (blue; residues 10–23), flap (dark yellow; residues 46–54), hinge (sky blue; residues 35–42 and 57–61), cantilever (orange; residues 62–78), α-helix (yellow; residues 86–93), and the 80s loop (boxed in black; residues 79–84). (**B**) HIV-1 PR heterogenous flap conformational states are represented as closed (dark gray; left), semi-open (yellow; center), and open (green; right), and are distinguished by distances between the Cα atoms of Asp25 and Ile50 (shown as spheres). This figure was generated using PyMOL Molecular Graphics Software, version 1.8, Schrödinger LLC. PDB ID: 3U71.

The PR enzyme is an obligate homodimer composed of two identical 99 amino acid monomers (Figure 3). The amino and carboxyl termini residues found at the dimer interface (residues 1–4 and 94–99) are conserved across all HIV subtypes [12]. Notably, the terminal F99 residue has been shown to play a crucial role in the formation of the functional homodimeric PR. Indeed, the F99A substitution leads to the formation of structured monomeric molecules, inhibiting dimerization and completely abolishing enzymatic activity [28]. The active site contains a conserved catalytic dyad (two Asp residues: one Asp residue contributed by each monomer) and a nucleophilic water molecule, which is characteristic of aspartyl PR [29]. Each monomeric subunit contributes an Asp residue to the catalytic dyad which is located at the bottom of the PR active site cavity (Figure 3). Entry of substrates or inhibitors into the active site is controlled by two β-hairpin loops termed the flaps (residues 46–54). The flap tips contain a conserved glycine-rich sequence, 46-MIGGIGGFI-54, which plays an important role in substrate recognition and binding [30,31]. The movement of these flexible flaps is largely aided by the hinge regions (residues 35–42 and 57–61) and regulate flap flexibility and stability. Opening of the flaps relies on the synergistic downwards motion of the cantilever (residues 62–78), fulcrum (residues 10–23), and hinge regions which also results in the rotation of each monomer about the β-sheeted dimer interface [14,32]. This combined movement is referred to as the hydrophobic sliding mechanism [33].

A dynamic image of the PR, revealed through NMR spectroscopy and molecular dynamics simulations, has revealed that the flaps exist in a dynamic equilibrium of heterogeneous flap conformations, namely, open, semi-open, and closed (Figure 3) [34,35,36]. The mobility of the flaps is critical for the function of the PR as the open conformation allows substrates or PIs to enter the active site cavity, while the closed conformation is responsible for the formation of intermolecular interactions with the substrate [30]. Traditionally, flap conformations are defined by measuring the distance between the alpha carbon (Cα) of the flap tip Gly51/Gly51′ residues, where interatomic distances < 7 Å, 7–12 Å, and >12 Å are defined as closed, semi-open, and open conformations, respectively. However, this definition is limited and can be affected by asymmetric movement of the flaps, as well as the occurrence of flap tip curling. As such, a more accurate description of these conformations is defined using the interatomic distance between the Cα of the catalytic Asp25/Asp25′ residues and the flap tip Ile50/Ile50′ residues [13,37]. Based on the new definitions, the conformations are consequently defined as interatomic distances of <17 Å, 17–22 Å, and >22 Å for the closed, semi-open, and open conformations, respectively (Figure 3).

### 1.4. Function of the HIV-1 Subtype C Protease

Understanding the structure of the PR active site and its mechanism of action has allowed for the design of PIs which target the active site and impede the function of the PR. Regardless of the viral PR subtype, HIV-1 PRs use a general acid–base reaction mechanism for the hydrolysis of the carbonyl carbon scissile bond on a substrate protein. Briefly, two Asp25 residues of the PR (one from each monomer) exist in opposing states of protonation and coordinate a water molecule that acts as a nucleophile during catalysis. When a substrate (e.g., Gag) is bound to the active site of the PR, the coordinated water molecule performs a nucleophilic attack on the carbonyl carbon of the scissile peptide bond, resulting in the formation of a tetrahedral intermediate enzyme–substrate complex. Protonation of the scissile amide nitrogen atom results in the breakdown of the intermediate complex and the subsequent release of the cleaved peptide products.

**Figure 4:**
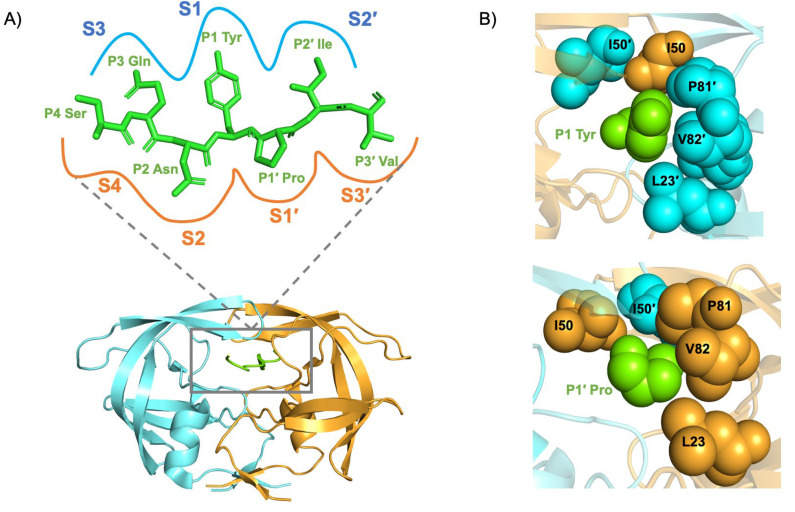
(**A**) Diagrammatic representation of the subsites of the HIV-1 PR active site and a Gag substrate. The subsites of the active site (gray box) are represented by S4–S1 and S1′–S4′ for those subsites toward the N- or C-terminus of the scissile bond, respectively. The PR monomers are colored blue and orange. The substrate sites are denoted similarly: P4–P1 and P1′–P4′. (**B**) The P1 and P1′ sites of the substrate fit into the S1 and S1′ subsites of the PR active site following a lock-and-key paradigm. This figure was generated using PDB ID: 3OTS.

**Figure 5:**
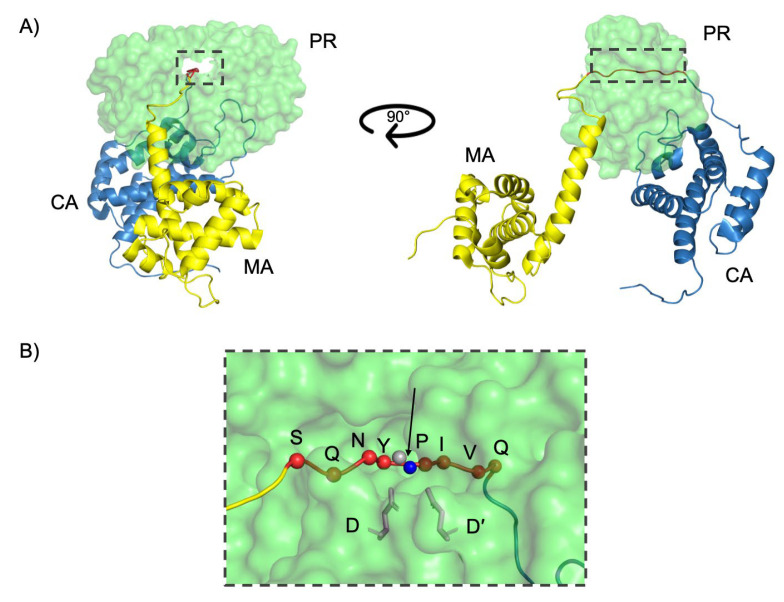
The function of the HIV PR. (**A**) PR (green) bound to the MA/CA cleavage site of the unprocessed Gag polyprotein (MA yellow, CA blue). The black box highlights the cleavage site being threaded through the active site cavity. (**B**) The MA/CA cleavage site heptapeptide (SQNYPIVQ; red spheres) is coordinated in the active site cavity. The catalytic aspartic acid residues (D/D′) are indicated as blue sticks. The scissile peptide bond is indicated by the black arrow and occurs between the P1 carboxyl carbon (gray sphere) of the and the P1′ amide nitrogen (blue sphere). This figure was generated using PyMOL Molecular Graphics Software, Schrödinger, LLC. PDB ID: 6I45 (PR); 1L6N (MA-CA polyprotein).

Structurally, the active site cavity of the PR is divided into eight subsites, each accommodating specific side chains of the substrate/inhibitor (Figure 4) [38]. The scissile peptide bond is positioned between the P1 and P1′ site of the PR (Figure 5). The amino acids positioned toward the N-terminus on the substrate are designated P1, P2, and P3 while those toward the C-terminus are termed P1′, P2′, and P3′ (Figure 4). Each side chain of the peptide (P3–P3′) binds a subsite formed by the PR residues, designated as S4–S3′. A maximum of seven residues expanding from S4–S3′ is needed for efficient catalysis [38].

### 1.5. Dynamics of the HIV-1 Subtype C Protease

In comparison to the amino acid sequence of the well-characterized HIV-1 subtype B PR, the C-SA PR contains eight naturally occurring amino acid polymorphisms (NOPs), Table 1 [11,12,31,32]. These polymorphisms are located outside of the PR active site region as follows: T12S, I15V, and L19I are found in the fulcrum region, M36I and R41K, in the hinge region, and H69K and L89M are found in the cantilever and 80s loop, respectively. Lastly, I93L is found in the α-helix of the PR monomer. The hydrophobic core of the PR contains 20 amino acids, namely, L5, V11, I13, V15, I19, A22, L24, I26, L33, L38, I62, I64, I66, V75, V77, I85, M89, L90, L93, and L97 [12,33]. Notably, four of the NOPs that occur in the C-SA PR sequence are located within the hydrophobic core of the PR, namely, V15, I19, M89, and L93. The remaining four NOPs (T12S, M36I, R41K, and H69K) are closely associated with core residues. The presence of NOPs in the C-SA PR has a profound effect on the hydrophobic sliding mechanism by altering the dynamics of the PR and, more specifically, the way the flaps change conformation over time [32]. In the subtype B PR, the cantilever and hinge regions move over I15 in the hydrophobic core, and this allows anchoring of the flaps. In the C-SA PR, however, the hydrophobic interactions within the core are altered due to the presence of the I15V, L19I, M36I, H69K, L89M, and I93L polymorphisms. Interestingly, the C-SA PR exhibits a higher propensity to sample open flap conformations when compared to the subtype B PR [12,39]. In addition, the C-SA PR has been shown to sample a wider variety of conformations exhibiting greater flap region conformational heterogeneity [40].

The hinge mutation, M36I, introduces an interesting effect on the flap dynamics of the C-SA PR. The E35-R57 salt bridge, which is adjacent to the M36I mutation, is the only salt bridge linking the flap and hinge regions of the subtype B PR and subtype C PR. Noticeably, the E35-R57 salt bridge of the C-SA PR is absent when observed via molecular dynamics simulations [32,41]. The E35-R57 salt bridge is thought to help lock the flaps into semi-open/closed conformations and its absence, therefore, contributes to the increased flap flexibility of the C-SA PR due to the M36I mutation altering the local geometry of the hinge region [32,41]. Furthermore, the L89M polymorphism is thought to increase the stability of the hinge, fulcrum, and cantilever regions of the C-SA PR due to the extensive contacts M89 forms with neighboring residues [42]. It is evident, therefore, that results from studies such as these indicate that there are significant dynamic differences between B and C viral PRs which cannot be ignored. Considering this, using a B PR as a representative PR of all subtypes, and designing drugs against this subtype, is not prudent. NOPs in the C-SA PR work synergistically to alter both the hydrophobic sliding mechanism and the flexibility of the flaps, resulting in the dynamic behavior of the C viral PR being markedly different in comparison to the B viral PR.

**Table 1:** Functional effects of NOPs in HIV protease.

NOP	Location in Protease	Functional Effects	References
T12S	Fulcrum	May influence local packing and backbone flexibility; proximal to hydrophobic core; and potential impact on flap dynamics.	[11,12,32]
I15V	Fulcrum/hydrophobic core	Reduces side-chain bulk, disrupts hydrophobic packing; alters anchoring of hinge/cantilever; and increases flap flexibility.	[11,12,33]
L19I	Fulcrum/hydrophobic core	Modifies core hydrophobic interactions and destabilizes fulcrum.	[11,12,33]
M36I	Hinge	Alters hinge region mobility; linked to increased flap conformational variability; and associated with drug resistance adaptation.	[12,32,43,44]
R41K	Hinge	Conserves charge but alters local hydrogen bonding and potential effects on hinge flexibility and dimer interface.	[11,12,31,32]
H69K	Cantilever	Increases basicity; affects electrostatics and flap-cantilever interactions; and promotes open flap conformations.	[11,12,39]
L89M	80s Loop/hydrophobic core	Subtle hydrophobic shift and influences PR core stability by increasing stability of hinge, fulcrum, and cantilever regions.	[11,12,40,42]
I93L	α-Helix/hydrophobic core	Conservative change; affects α-helix packing; and may influence flap anchoring and hydrophobic sliding mechanism.	[11,33,39]

## 2. HIV-1 Protease Inhibition

There are currently nine PIs approved for antiretroviral therapy (ART) by the FDA, namely, amprenavir (APV, PubChem CID: 65016), atazanavir (ATV, PubChem CID: 148192), darunavir (DRV, PubChem CID: 213039), indinavir (IDV, PubChem CID: 5462355), lopinavir (LPV, PubChem CID: 92727), nelfinavir (NFV, PubChem CID: 64142), ritonavir (RTV, PubChem CID: 392622), saquinavir (SQV, PubChem CID: 60787), and tipranavir (TPV, PubChem CID: 54682461). All PIs directed at the subtype B PR exhibit relatively uniform intermolecular contacts with the active site, and function as competitive inhibitors by competing with the substrate for the active site of the PR [45]. PIs are specifically designed to mimic the tetrahedral transition-state observed during the proteolytic reaction [46]. The process of inhibitor binding shifts the PR flaps into the closed conformation where the flaps exhibit significantly reduced mobility.

Due to the highly specific configuration of the transition-state, transition-state analogs exhibit binding affinities for the PR active site that are in the picomolar range (pM) [47,48,49]. Together with the presence of a non-hydrolyzable hydroxyethylene core, PIs exhibit higher specificity for the PR than their corresponding substrates and inhibit the reaction mechanism of the PR [47,48,49]. However, due to the selective pressure imposed by PIs in conjunction with the mutation-prone nature of the viral RT, the virus rapidly accumulates sequence polymorphisms which favor inhibitor resistance. Indeed, amino acid polymorphisms associated with reduced drug susceptibility are observed at over half of the positions of the PR sequence [50].

### 2.1. NOPs, Drug-Induced Protease Mutations, and Inhibitor Resistance

The paradigm describing the accumulation of drug resistance mutations in the PR is centered around the presence of two distinct, but equally relevant, types of mutations. Generally, it is believed that resistance begins with the onset of primary mutations, which refers to amino acid substitutions that directly impact drug susceptibility. Most often, primary mutations occur directly within the active site cavity and affect drug binding through altering the geometry of the drug binding interface [51,52]. Since PIs are smaller and more rigid molecules relative to their substrate counterparts, inhibitors are unable to account for changes in local geometry of the interface. Therefore, primary mutations reduce drug binding affinity through altering the geometric complementarity between the active site and inhibitor. However, primary mutations still possess the potential to decrease the binding affinity of the natural substrates and, therefore, the PR sequence accumulates compensatory mutations in the hydrophobic core of the PR as well as in the Gag/Gag-Pol cleavage sites [53]. These non-active site mutations, or secondary mutations, are thought to compensate for the geometric perturbations caused by primary mutations in a manner that maintains the catalytic efficiency of the PR [54]. Together, the accumulation of complex combinations of primary and secondary mutations results in resistance through compromising inhibitor binding while maintaining selectivity for the naturally occurring substrate molecules.

In some cases, NOPs that occur in non-B subtypes are resistance-associated mutations with respect to the subtype B PR. The key role that NOPs can play in reducing the PI susceptibility of non-B subtypes is highlighted from thermodynamic analyses of the binding of FDA-approved PIs between the subtype B and subtype C PR. These analyses have revealed that PIs bind to the subtype C PR with 3- to 24-fold weaker binding affinity in comparison to the subtype B [55]. Interestingly, when considering the subtype C PR, it has been shown that the NOPs present in the subtype C increase the propensity of the apo PR to adopt the open flap conformation [32,40]. Moreover, analysis of subtype C PR flap tip curling indicates that in some cases the inhibitor-bound complexes, such as ATV, DRV and TPV, remain in the semi-closed conformation and do not achieve the fully closed conformation [12,13,32,39]. Non-active site mutations have been shown to increase the mobility of the flap regions when in complex with inhibitors, suggesting that such mutations prevent PIs from successfully “locking down” the PR active site into the closed conformation [13,38].

As mentioned previously, substrate and inhibitor recognition relies on the motion of the PR flap regions. Moreover, the hydrophobic sliding mechanism illustrates that the flap regions exist in a delicate equilibrium that is maintained, at least partially, by the rearrangement of hydrophobic residues in the core of the PR. As such, it is postulated that mutations in the core of the PR, whether drug-induced or naturally occurring between subtypes, may alter the packing of the core due to changes in the size and shape of the mutated residues. The mechanism of action of resistance-conferring mutations that occur outside of that active site is not well-understood. However, it has been suggested that their presence in the PR sequence alters the dynamic nature of the enzyme, particularly the flap regions [14,33,56,57]. Since mutations of this nature are not located inside the active site, steric irregularities are not likely to be responsible for their mechanism of action in reduced inhibitor binding affinity. Rather, it is more likely that their presence alters the conformational equilibrium of the PR flaps, resulting in novel conformational heterogeneity in PI-resistant PR variants [14,33,57]. Therefore, when considering the inhibition of non-B subtypes, it is likely that observable decreases in drug susceptibility to PIs can be attributed to the altered dynamics and flexibility of the flap regions of the PR. This fact is mirrored when considering the requirement for constellations of non-active site mutations to accumulate in multi-drug resistant (MDR) PR variants. It is clear that the occurrence of several non-active site mutations alters the conformational equilibrium of the PR flaps and induces novel conformational heterogeneity in PI-resistant PR variants [13,14,56,57,58]. It is evident, therefore, that drug resistance arises, at least partially, from alterations in the conformational equilibrium of the flap regions [14,59]. Therefore, differences in drug binding affinity are not only explicable due to the presence of NOPs in non-B subtypes but, rather, these inherent differences are explicitly expected to directly impact drug binding. It is prudent to consider the mechanistic details when designing the next generation of PIs.

In conjunction with the mutations that accumulate in the PR sequence, the Gag and Gag-Pol polyproteins have been seen to accumulate sequence mutations that have been implicated in reduced drug susceptibility. This observation suggests cooperativity between the PR and Gag/Gag-Pol mutations in order to obtain high levels of resistance [21]. Drug resistance is caused by polymorphisms that alter the balance of recognition to favor the substrate over the inhibitor. Often mutations that confer drug resistance result in a decreased replication capacity which is compensated for by mutations in the Gag sequence. This compensatory mechanism has been demonstrated through the co-evolution of the Gag substrate, which can restore the functional phenotype of a mutated PR to that of the wild-type (WT) [60]. Another study demonstrated that a mutated PR exhibited susceptibility to PIs when paired with the WT Gag protein sequence. However, susceptibility was rescued with the presence of the mutant’s naturally occurring Gag sequence, which contained mutations in its cleavage sites [61]. Therefore, while mutations in the PR reduce inhibitor affinity, efficient substrate processing is maintained through this co-evolutionary adaptation of Gag [62]. Importantly, substrate specificity is not due to a specific amino acid sequence, but rather by a conserved three-dimensional shape, referred to as the “substrate envelope”, which represents a consensus volume formed by the packing of substrate residues. This envelope facilitates recognition by the PR through spatial rather than sequence constraints, resulting in interdependent substrate contacts [62,63]. Consequently, drug resistance mutations in the PR that negatively affect recognition of one substrate position are often compensated by secondary mutations at other positions within the Gag substrate. This interdependency has been substantiated by multiple studies demonstrating co-evolution between Gag cleavage site mutations and PR variants, or vice versa [20]. It is noteworthy that the emergence of novel mutations in the PR sequence has been decreasingly reported, an observation that suggests that a limited number of mutations are tolerable within the PR sequence itself. However, the Gag polyprotein is approximately 500 amino acids in length, suggesting that the polyprotein would be more tolerant of a higher number of compensatory mutations in its sequence. Although the mechanism through which mutations in the Gag sequence contribute to PI resistance has yet to be fully understood, it is thought that these mutations work synergistically to simultaneously reduce intermolecular contacts with PIs while maximizing substrate binding.

Gag mutations play a critical role in the development of PI resistance in HIV-1, particularly in subtype C, where they contribute to viral fitness and reduced drug susceptibility. These mutations can arise at both cleavage sites and non-cleavage sites within the Gag protein, often functioning independently or synergistically with PR mutations to confer resistance [53,64,65]. Mutations in both regions frequently emerge together, allowing the virus to adapt under antiretroviral pressure while maintaining structural integrity and replication capacity [16,21,66]. In subtype C, distinct co-mutational patterns have been observed. The L76V mutation in the PR paired with specific Gag cleavage site mutations significantly impacts drug binding and resistance [16] while the A431V mutation in Gag has been shown to act in concert with PR mutations, enhancing resistance mechanisms and supporting viral survival [21,67]. Importantly, Gag mutations can alter PI susceptibility even in the absence of major PR mutations, underscoring the need to include full-length Gag analysis in resistance assessments [65]. Non-cleavage site mutations also play a crucial compensatory role by restoring viral fitness compromised by PR mutations. Subtype C-specific patterns have been identified, including Gag mutations at positions 373, 374, 378, 428, 431, 449, 451, and 453, which frequently emerge in patients failing PI-based therapy without accompanying major PR mutations [65]. The functional interplay between Gag and PR mutations is pivotal for preserving the cleavage efficiency and maturation of the virus, both of which are essential for infectivity [21,66]. Understanding this co-evolution of Gag and the PR and the resulting resistance patterns in subtype C is vital for optimizing therapeutic strategies and developing effective resistance management approaches [16,21,65,67].

### 2.2. Effect of Insertion Mutations on the Dynamics of HIV-1 Subtype C Protease

**Figure 6:**
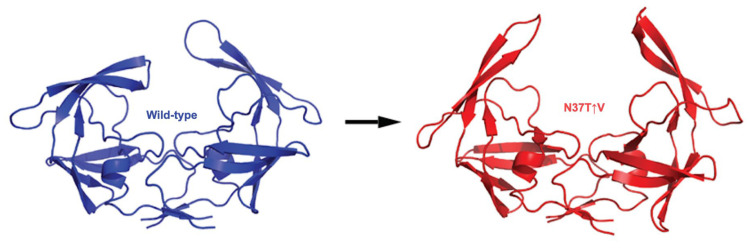
Comparison of WT (blue) and N37T↑V (red) hinge and flap dynamics. The fully open conformation of the N37T↑V flap region opens to a greater extent than the WT PR due to the increased flexibility of the hinge region due to the N37T↑V mutations. Image obtained from reference [41]. The molecular dynamics trajectory coordinates are provided as Supplementary Materials.

Insertion mutations are a rather rare, but not insignificant, type of mutation that occurs in the PR sequence. Amino acid insertions rarely occur in non-B HIV-1 subtype strains and do not play a major role in drug susceptibility, only contributing to drug susceptibility in the presence of additional mutations [68,69]. Amino acid insertions in the C-SA PR gene have been observed in treatment-naïve patients with a prevalence of 2.5% [69,70]. These insertions commonly occur in the hinge region, resulting in changes to the active site pocket and flap mobility, and could be involved in reduced drug susceptibility by altering binding affinities of PIs [31,71,72]. A hinge region insertion C-SA PR mutant, N37T↑V, was isolated from a South African PI-naïve infant [31]. In order to denote a mutation that involves the insertion of an amino acid, the “↑” notation was devised by Sayed and Achilonu. In addition to a mutation and insertion at codon 37 in the hinge region, N37T↑V contains ten background mutations, namely, I13V, G16E, I36T, P39S, D60E, Q61E, I62V, L63P, V77I, and M89L. Often, changes occurring in the hinge region of the PR directly affect flap dynamics since the hinge is associated with the movement and flexibility of the flaps. Consequently, N37T↑V displays a modification in flap and hinge region dynamics, with the flap region achieving a wider opening of the flap regions (Figure 6) [41]. This wider flap conformation can be correlated to an increase in the curling of the cantilever tip residues, I67/C68/G69, with the flap region fluctuating more readily when complexed with PIs [73]. Additionally, the hinge region mutations of N37T↑V results in excessive flexibility of the hinge which could be detrimental and is compensated for by stabilizing effects contributed by the background mutations [73]. A different hinge region mutant, containing a mutation and insertion at codon 36, was discovered in a HIV-positive mother who was treated with RT inhibitors but was PI-naïve. This mutant, termed I36T↑T, contained three additional background mutations, namely, P39S, D60E, and Q61E. The I36T↑T hinge region mutant exhibited increased flap flexibility and movement in the presence of nelfinavir, resulting in more semi-open conformations of the complex [74]. Another hinge region mutant, L38↑N↑L, isolated from a drug naïve-infant whose mother was exposed to RT inhibitors, has been identified and contains a subset of mutations—K20R, E35D, R57K, V82I, and a double insertion of asparagine and leucine in the hinge region at position 38. Consequently, these insertions increased the structural stability and flexibility of this PR with a flap shift to a predominantly closed conformation, implicating a possible mechanism for drug resistance [75]. It is highly likely that the prevalence of insertion mutations will increase and, therefore, the effects of these insertions on the PR needs to be explored.

### 2.3. Clinical Studies Demonstrating Reduced PI Efficacy in Subtype C

Clinical studies report that up to 60–67% of patients on second-line PI regimens in subtype C-dominant regions such as South Africa experience virological failure within two years [76,77]. Notably, major PI resistance mutations are often absent in 50–70% of these cases, suggesting that factors beyond classical resistance, including adherence challenges and subtype-specific polymorphisms, significantly contribute to treatment failure [76,77]. Polymorphisms such as M36I and L63P, present in over 90% of subtype C viruses, have been linked to reduced PI susceptibility and facilitate the emergence of drug resistance [76,78]. These findings indicate that standard PI regimens, designed mainly based on subtype B data, may be less effective in subtype C patients and highlight the urgent need for improved resistance monitoring and the development of subtype-specific treatment strategies.

## 3. Novel Paradigms for Drug Development and PR Inhibition

Despite the propensity for HIV-1 to acquire resistance to antiretroviral agents, PIs have generally been known to exhibit a high genetic barrier to resistance in the context of subtype B and subtype C infections [79]. The accumulation of resistance-conferring mutations in the PR due to prolonged exposure to clinically available PIs has been well-documented [80,81]. Indeed, the accumulation of such mutations in the virus is the leading cause for therapy failure. These issues are compounded by the adverse side effects of antiretroviral regimens, which result in the discontinuation of treatment. Thus, there remains a need for the development of novel antiretroviral drugs with better features such as greater potency, a higher genetic barrier to resistance, and minimal or no side effects. Moreover, the role of NOPs in the resistance mechanism of non-B subtypes of HIV illustrate the need for effective drugs across subtypes.

### 3.1. Darunavir Scaffold-Based Next-Generation Inhibitors

Darunavir, the latest PI to be approved for clinical use, maintains a high genetic barrier to resistance development. This means that DRV maintains inhibitory activity against WT subtype B PR and resistant PR variants that are no longer susceptible to some older PIs. In the context of the subtype C PR, DRV has been shown to exhibit decreased affinity due to the presence of NOPs [55]. However, the potency of DRV has still been shown to be greater when binding to the WT subtype C PR in comparison to when it is in the presence of several resistance-conferring mutations [32,43,82].

**Figure 7:**
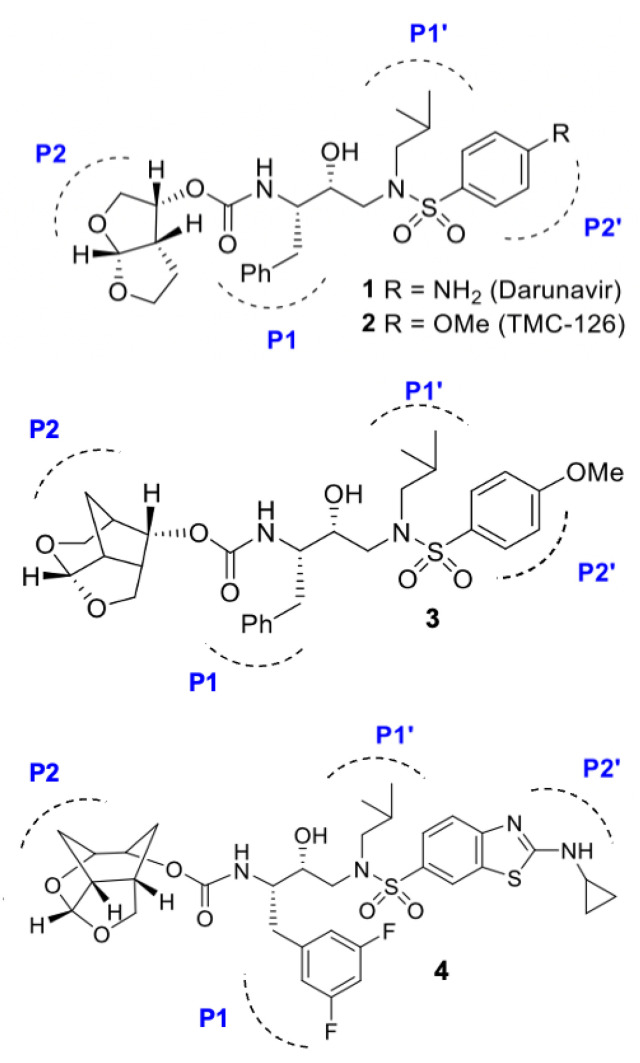
Structures of HIV PIs 1–4. Potent PIs have been developed based on the structural scaffold of darunavir (**1**), the most recent inhibitor to be approved for clinical use. Novel inhibitors (**2**–**4**) [76,77,78] are generated through modifying the moieties present at the P1, P2, and P2′ subsites of darunavir.

It is thought that the basis for the high genetic barrier to resistance that is exhibited by DRV is largely due to the formation of hydrogen bonds between the PI and the backbone atoms of the PR active site [83]. Specifically, the oxygens of the bis-tetrahydrofuran (bis-THF) moiety of DRV at the P2 ligand site (Figure 7, inhibitor 1) form strong hydrogen bonds with the amide group of the PR S2 subsite [83]. Designing inhibitors that interact with the backbone of the PR has been the basis of development for novel PIs. This is because bonds formed with the PR backbone are less likely to be affected by side chain mutations and, therefore, would be less susceptible to resistance-causing active site mutations. The X-ray structure of the subtype B PR in complex with DRV reveals that the P2′ ligand (4-aminosulfonamide) is involved in water-mediated hydrogen bonding with the backbone amide NH of Gly48′ through two crystallographic water molecules [84]. Based upon this observation, it has been hypothesized that modifying the P2′ ligand site to replace one of these two crystallographic water molecules may assist in maintaining strong hydrogen bonds with backbone Asp30 and may potentially form a water-mediated hydrogen bond with the backbone of Gly48′, which is located in the flap region of the PR [85,86].

The basis of developing next-generation PIs that bind optimally with the backbone of the PR active site has, therefore, been largely driven by developing derivative PIs based on the scaffold of DRV and its derivative, TMC-126 (Figure 7, inhibitor 2). New inhibitors have been designed by incorporating more complex chemical structures derived from modifications on three moieties of the DRV scaffold, namely subsites P1, P2, and P2′. Several novel PIs with high potency have been reported that incorporate a variety of heterocyclic P2 ligands to enhance interactions with the S2 subsite [87,88,89,90], with the intention of increasing hydrogen bonding potential with Asp29 and Asp30.

Additionally, the optimization of both P1 and P2′ ligand moieties has proven to improve potency through improving the van der Waals interactions formed with the S1 and S2′ subsites, respectively [89,90,91]. As such, simultaneous optimization of both the P1, P2, and P2′ structural templates of DRV have been proposed for highly potent PIs [91]. The incorporation of a novel crown-like tetrahydropyrano–tetrahydrofuran (crown-THF) as the P2 ligand (Figure 7, inhibitor 3) has been shown to promote additional van der Waals interactions in the active site, and these PI derivatives were seen to maintain potent antiviral activity against MDR PR variants [92,93,94]. Such crown-THF inhibitors have been shown to maintain potent inhibitory activity against MDR subtype B variants through improved ligand-binding site interactions [92,95]. More recently, a new class of PIs containing cyclohexane-fused bistetrahydrofuran (Chf-THF) as the P2 ligands in combination with (R)-hydroxyethylaminesulfonamide isosteres as the P2′ ligands have shown promise for next-generation inhibitors of the PR (Figure 7, inhibitor 4). This inhibitor has been shown to maintain exceptional antiviral potency against a panel of highly MDR subtype B variants. This new design shows promise for hydrogen bonding interactions with the backbone amide groups of Asp29 and Asp30, while the additional methylene groups around the bis-THF exhibit better van der Waals interactions with the S2 subsite. The potency of such novel inhibitors has not been tested in subtype C; however, the panel of highly MDR subtype B variants tested contain the resistance-associated mutations M36I/I93L, which are resistance-associated NOPs in subtype C. As such, it may be suggested that novel inhibitors containing crown-THF and Chf-THF moieties could possess the potential to maintain potency across subtypes.

### 3.2. Dual Action Inhibitors of HIV Reverse Transcriptase and Protease

**Figure 8:**
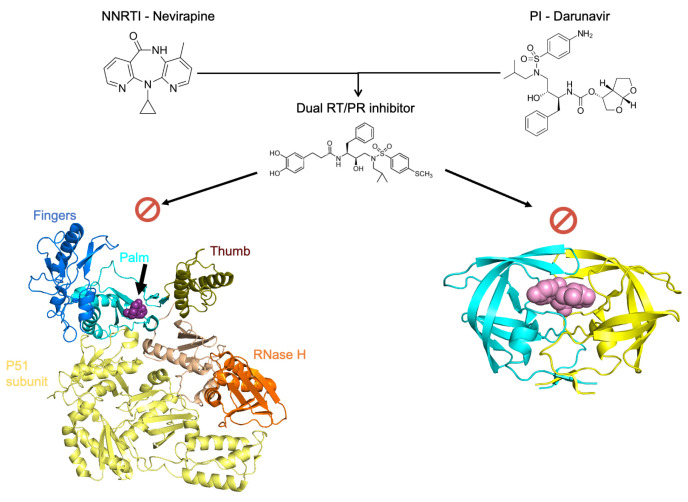
Dual inhibition of HIV RT and PR enzymes. The structures of an NNRTI and PI are shown as well as a novel dual RT/PR inhibitor (compound 38c, [84]). The viral RT is divided into subdomains, namely, fingers (blue), palm (green), thumb (orange), and an RNAse H domain (red). The black arrow indicates the location of the NEV ligand (yellow) in the DNA polymerization active site region of RT. The PR dimer is shown in complex with DRV (pink) with monomers colored cyan and yellow. This figure was generated using PyMOL Molecular Graphics Software, version 1.8, Schrödinger, LLC. PDB ID: 1JLF (RT), 4DQB (HIV PR dimer).

**Figure 9:**
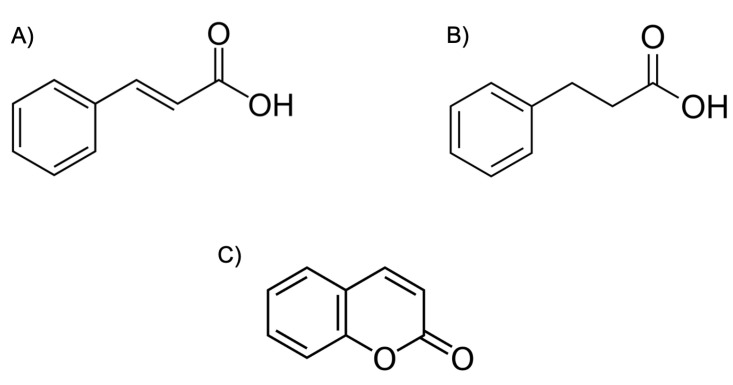
Structure of (**A**) cinnamic acid (PubChem CID: 444539), (**B**) phenyl-propionic acid (PubChem CID: 107), and (**C**) coumarin (PubChem CID: 323).

The design of inhibitors that are capable of simultaneously inhibiting the action of the RT and PR represent a promising inhibitory paradigm. Potent dual inhibitors could radically change drug treatment regimens through minimizing the need for several drug compounds targeting various enzymes in the life cycle of HIV. Dual action inhibitors could decrease the pill burden on HIV-positive individuals and decrease the extent of adverse physiological side effects. Together, these factors may increase the therapeutic compliance of those undergoing ART, thereby reducing the discontinuation rates of treatment. The difficulty with designing dual-action RT/PR inhibitors lies in the differences in the structure and function of these two enzymes. For instance, the HIV-1 RT is an asymmetric heterodimer composed of a 560 amino acid subunit (p66) and a 440 amino acid subunit (p51) [96]. The p66/p51 RT heterodimer possesses two active sites (Figure 8), the DNA polymerization active site and the RNase H active site, both of which reside in the p66 subunit (Figure 8). The p66 contains several subdomains, namely, the fingers (residues 1–85 and 118–155), palm (residues 86–117 and 156–237), and thumb (residues 238–318) [96]. Moreover, the structure of NNRTIs, NRTIs, and PIs differ structurally and chemically (Figure 8). However, there is evidence to suggest that the synthesis of inhibitors with dual action is feasible. Recently, coumarin derivatives were shown to exhibit moderate binding affinities (µM) to both the PR and RT enzymes of the subtype B [97] and subtype C [98]. In an effort to develop more potent dual inhibitors, the following chemical changes were introduced: (i) To better fit the spindly binding pocket of RT, cinnamic acids or phenyl-propionic acids (Figure 9) with long linear linkers were used instead of coumarins. (ii) Aromatic rings were maintained in the P2 ligand site in order to optimize π–π interactions with both the PR and RT. (iii) Finally, aliphatic chains or rings were introduced in order to better adapt to both active site cavities [99].

Notably, these newly synthesized inhibitors showed an increase in anti-RT activity by one or two orders of magnitude while maintaining potency against the PR. For example, compound 38c (Figure 8) exhibited IC50 values of 2.02 nM and 0.43 mM for the PR and RT, respectively, which are comparable to positive control inhibitors and DRV and EFV exhibit IC50 values 1.52 nM and 0.091 mM for the PR and RT, respectively. This novel class of inhibitors illustrates that dual RT/PR inhibitors can be designed to simultaneously target the active site of both enzymes. The basis for targeting both ligand binding sites of RT and PR is thought to be due to the shared nature of interactions and the uniformity of key residues shared by the active sites [99]. Molecular docking studies revealed that the dual inhibitory potency was largely due to the presence of an aromatic ring as well as hydroxy groups at the P2 ligand site, which were crucial for the formation of hydrogen bonds and van der Waals interactions with both PR and RT.

### 3.3. Novel Fullerene Derivatives as Dual RT/PR Inhibitors

Fullerene (also called buckminsterfullerene or buckyball) is a carbon allotrope with pharmaceutically useful properties based on its three-dimensional structure. However, evaluating the bioactivity of fullerene is limited due to poor water solubility. As such, fullerene derivatives with hydrophilic moieties have been developed to study its potential bioactivity and usefulness. Interestingly, the pharmacological properties of fullerene are broad and include antibacterial activity [100] and inhibition of various enzymes including the HIV RT [101] and HIV PR [101,102]. With regard to the usefulness of fullerene derivatives as anti-HIV agents, several fullerene-based compounds have been reported to exhibit dual inhibitory potential for RT and PR. In particular, fullerene derivatives bearing a hydroxy–methyl–carbonyl (HMC) moiety have been shown to bind favorably with the active site aspartic acid residues of the PR during docking simulations [103]. This is due to the HMC moiety mimicking the transition-state isostere of substrate processing in a manner that is similar to the inhibitory mechanism of clinically available PIs. A study of fullerene derivatives containing a pyrrolidine ring and an alkyl chain linker to the HMC moiety exhibited potent PR inhibition properties in the low micromolar (μM) range [101]. Although the inhibition activities of these fullerene derivatives were weaker than the clinically available PI ritonavir, cell-based assays revealed that these compounds exhibited membrane permeability comparable to that of RTV and showed no indication of serious cytotoxicity. Thus, the suitability of fullerene-based derivatives as drug candidates for dual inhibition of RT/PR shows promise for the development of novel drug candidates which could be used to improve anti-HIV therapy.

### 3.4. Allosteric Inhibitors of the HIV Protease

**Figure 10:**
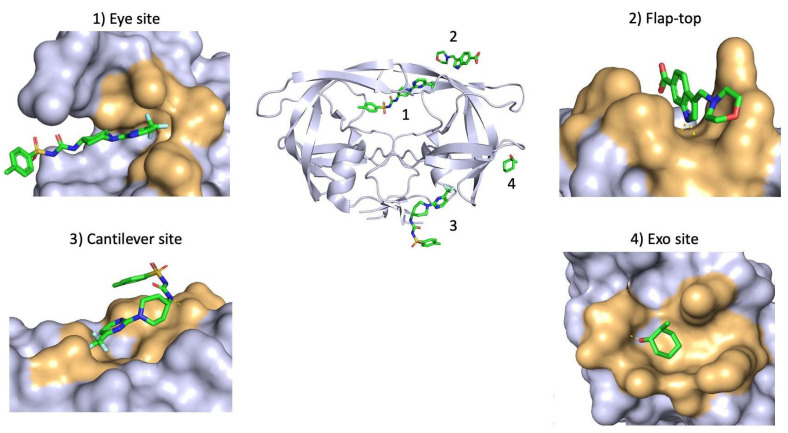
Allosteric binding sites identified for the HIV PR. Four allosteric binding sites for small molecules have been identified on the surface of the PR, namely, the Eye site (**1**) (PDB ID: 5VJ3), the flap-top pocket (**2**) (PDB ID: 4TVG [109]), the cantilever site (**3**) (PDB ID: 5W5W), and the Exo site (**4**) (PDB ID: 3KFN [107]).

Allosteric inhibitors are small molecules that bind to the enzyme at an allosteric site, which is not at the same location as the active site, in a manner that affects the activity of the enzyme through modulating its shape, or possibly, its dynamics. The design of allosteric inhibitors of the PR assumes that the global dynamic nature of proteins is conserved, and so interfering with the dynamic equilibrium may interfere with the function of the enzyme. Indeed, it has been postulated that disturbances to this dynamic equilibrium are the basis of many non-active site resistance mutations [14]. Indeed, molecular dynamics simulations have illustrated that the conformational dynamics of drug-resistant PR variants are different from that of the WT PR [104,105]. Notably, a recent study combining machine learning and molecular dynamics simulations illustrated that drug-resistant PR variants converge to a similar feature phenotype, suggesting that drug resistance is mediated through specific mechanisms that are independent of the site or type of mutations [106]. As such, allosteric inhibitors that interfere with the global dynamics of the protein represent an encouraging prospect for future therapies. Allosteric inhibitors that are able to shift the conformational equilibrium of MDR PR towards behavior that resembles a more WT-like state may prove to be an effective course of action. The potency of such allosteric inhibitors has yet to be fully realized; however, the co-administration of allosteric inhibitors alongside traditional PIs may prove to be a promising model of inhibition. To date, four allosteric binding sites have been identified for the dimeric PR, namely, the Exo site, flap-top pocket, Eye site, and Cantilever site (Table 2). Several small molecules have been shown to bind to these sites (Table 2, Figure 10) and, interestingly, crystal structures have revealed that existing PIs, APV and DRV, may bind to the flap-top pocket.

The exact mechanism of action for the allosteric inhibition of the PR is not fully elucidated; however, some experimental evidence suggests that the modulation of these sites alters the mobility of the flaps. For instance, the Glu35-Arg57 salt bridge forms part of the flap-top pocket and has been shown to play an important role in the conformational stability of the flap regions [32,41]. As such, allosteric inhibitors that bind to the flap-top pocket may modulate the stability of the flaps via the Glu35-Arg57 salt bridge. Curling of the flap tips into the Eye site is thought to drive the opening of the flap regions [113], and as such blocking the Eye site is hypothesized to prevent curling of the flap tips in a manner that prevents the opening of the flaps to accommodate substrates. Alternatively, due to the location of the Eye site, allosteric inhibitors may prevent the flaps from closing fully which could negatively impact the catalytic efficacy of the PR. Either of these modes of binding provide a possible mechanism of action for inhibitors designed to target the Eye site of the PR through altering the dynamic equilibrium of the flaps in a manner that diminishes substrate binding and catalysis. More recently, the curling of the tip regions of the cantilever have been hypothesized to play a significant role in the functional dynamics of the PR [13]; therefore, allosteric inhibitors that bind to this site and modulate the mobility of the cantilever tips could represent a novel mode of inhibition of the PR. There is evidence to suggest that co-administration of allosteric regulators, such as 1F1, 4D9, and Br6, restores the potency of clinically available PIs against MDR PR variants by restoring a more WT-like behavior [110,113,114]. As such, when considering the importance of the global dynamic nature of the PR in its functionality, the use of allosteric inhibitors that complement the effect of active site inhibitors could restore drug potency against MDR HIV variants.

### 3.5. Biologics Targeting HIV Protease

#### 3.5.1. RNA Aptamers

Aptamers are a class of short, single-stranded RNA or DNA oligonucleotides that can form higher-order three-dimensional structures and possess high binding affinity (nM-µM) and selectivity for target molecules [115]. These target molecules include proteins, peptides, toxins, small molecules, carbohydrates, and whole cells. Such aptamers possess several characteristics that are advantageous for therapeutic purposes. Aptamers are smaller in size than other biologics, such as antibodies, but maintain selectivity and binding affinity that is comparable to their antibody counterparts. Unlike antibodies, aptamers can be synthesized in bulk with relative ease, and they can be chemically modified to improve their pharmacokinetic and pharmacodynamic properties for specific tasks. Anti-HIV PR RNA aptamers represent a unique class of potent inhibitors in anti-HIV therapy. RNA aptamers are able to bind to the PR and inhibit its proteolytic activity through non-competitive inhibition. The binding locations of RNA aptamers can be varied; however, since allosteric inhibition sites have been described for other small molecules it is thought that aptamers will successfully inhibit the PR through binding with the hinge region, flap-top pocket, flap tips, and dimerization interface. Since aptamers are non-peptidic inhibitors, the goal of their design is interference with the dynamic equilibrium of conformations that the PR is known to sample. As such, aptamers are likely to bind to non-active site regions of the PR. However, aptamers may also prove to be effective dimerization inhibitors which represent a different inhibition mechanism which renders the enzyme inactive due to its obligatory dimeric nature. Since active site inhibitors target the dimer and are susceptible to resistance mutations in the active site, it is suggested that dimerization inhibitors may overcome this limitation through interacting with the dimer interface of the PR.

**Figure 11:**
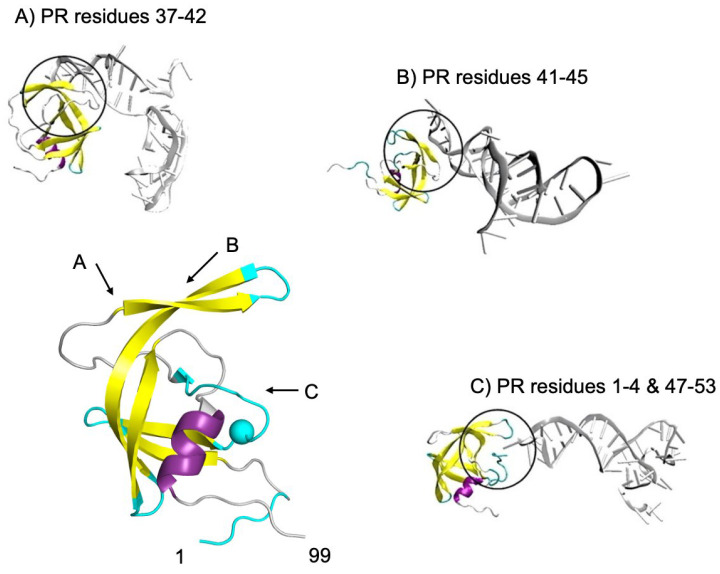
Sites of interaction between the PR monomer and three different oligonucleotide aptamers. Complex (**A**) is formed when the aptamer makes contact with the PR hinge and flap region residues (37–42), complex (**B**) forms with PR residues 41–45 of the flap region, while complex (**C**) makes contact with the N-terminal residues (1–4) and residues of the flap tip (47–53). The active site Asp25 residue is indicated by a cyan sphere. PDB ID: 1HPP, methodology available in reference [116].

Examples of oligonucleotide aptamers are shown in Figure 11, which illustrates the complex structures of three aptamers bound to different regions of the PR monomer. Molecular dynamics simulations of these complexes revealed that aptamer complex A exhibited decreased flexibility in the hinge region of the PR [116]. In this complex, the aptamer forms interactions with residues of the hinge region and stabilizes their movement. The flexibility of the hinge region is known to play a crucial role in the conformational freedom exhibited by the flap regions of the PR [14,33,59]. Therefore, locking the hinge into a more stable conformation has been hypothesized as a method of preventing the function of the PR by restricting the conformational changes required for catalysis. The aptamer complex B (Figure 11) illustrated an increase in the global dynamics and flexibility of the PR backbone. This indicated that the binding of the aptamer to this location which forms part of the “flap-top” pocket of the PR serves to destabilize the PR, which suggests that the dynamic equilibrium of the PR has been altered by this interaction. Complex C (Figure 11) revealed another form of inhibition through interacting with the dimer interface of the monomeric PR. The ability of the aptamer to form stable interactions with the dimer interface would likely inhibit dimer formation and, therefore, the function of the PR. The ability of RNA aptamers to bind to various allosteric locations on the PR illustrates the diverse potential for the inhibitory function of these molecules in anti-HIV therapy.

#### 3.5.2. Peptide-Based Inhibitors

**Figure 12:**
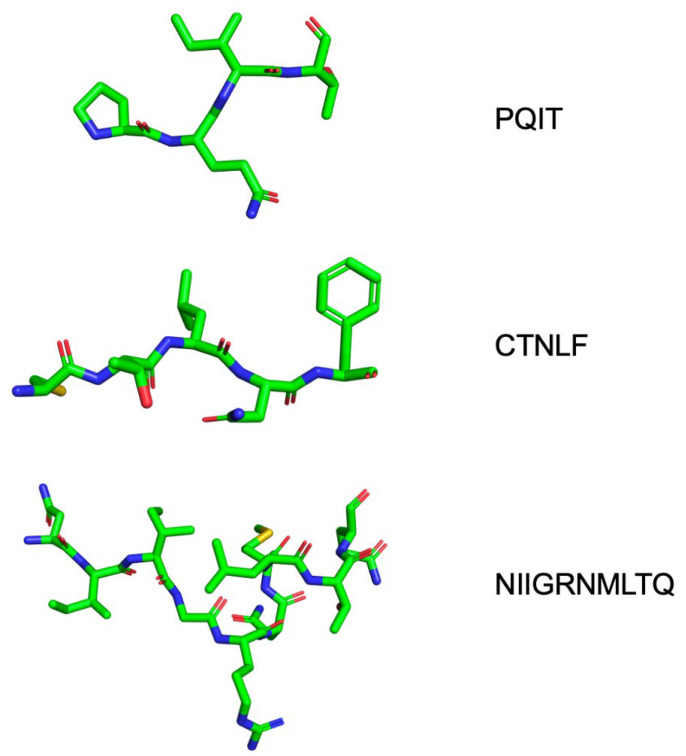
Peptide biologics designed to inhibit HIV-1 PR. Novel peptide biologics are designed using segments of the subtype B PR sequence as a template. Thermodynamic inhibitors resemble sections of the hydrophobic core of the subtype B PR (NIIGRNMLTQ) and disrupt its native structure by penetrating into and interacting with the hydrophobic residues in the core. Dimerization inhibitors are able to bind to and inhibit dimerization by mimicking the peptide sequence that comprises either side of the dimerization interface (PQIT/CTNLF).

Several peptide inhibitors of HIV PR have been identified experimentally [117]. While active site mutations can alter the shape of the active site of the PR, peptides have shown great promise as novel PIs because of the versatile range of locations that are targetable by peptides other than the catalytic domain. For example, peptides synthesized to be complementary to the N- or C-terminus of the PR (Figure 12) can successfully inhibit dimerization and, therefore, the activity of the PR through binding to the dimerization interface [118,119]. Perhaps most interestingly, peptide inhibitors have been suggested as potential inhibitors of the PR through shifting the thermodynamic equilibrium of the protein towards a denatured state [120]. Such inhibitors are designed to shift the thermodynamic equilibrium of the protein towards a denatured state and, in general, escape the straightforward paradigm of competitive inhibition and resistance mutations. These peptide inhibitors affect the conformational properties of the PR monomer and shift its thermodynamic equilibrium towards a state in which the active site is not formed, resulting in an inactive enzyme [120]. Peptides with the same sequence as segments of the hydrophobic core of the PR (Figure 12) have been suggested to successfully disrupt the PR monomer. Such folding inhibitors represent a novel and potentially powerful method of inhibiting the HIV PR enzyme [121,122]. In order for peptide-based inhibitors to be effective, their sequence must not resemble that of the natural Gag/Gag-Pol cleavage sites. As such, peptide-based biologics exhibit a form of non-competitive/allosteric regulation of the activity of the PR.

## 4. Concluding Remarks

HIV/AIDS continues to pose a significant socio-economic burden on developing countries, particularly in sub-Saharan Africa. Approximately 39 million individuals are HIV-positive globally, with 70% of infections occurring in sub-Saharan Africa. Currently available FDA-approved PIs used for antiretroviral therapy were designed using the HIV-1 subtype B PR as a template. The HIV-1 subtype B occurs mainly in North America and Western Europe, and, as a result, the majority of global research has focused on this subtype. Moreover, rational drug design approaches have not focused on the HIV-1 subtype C which, shockingly, accounts for more than 50% of global infections. There is evidence to suggest that FDA-approved PIs show diminished efficacy in the context of non-B PR subtypes. The significant genetic diversity of HIV means that substantial structural and behavioral differences are present at a fundamental level when comparing different HIV-1 subtypes. Such differences are likely to affect the efficacy of current drug regimens in non-B subtypes. Future research and drug development efforts must focus on the design of inhibitors that are effective across subtypes. Detailed structural, biochemical, and biophysical studies are required to produce novel drug molecules that possess high potency towards HIV-1 subtype-specific protein targets.

## Figures and Tables

**Table 2:** Allosteric binding sites of the HIV PR and the associated molecules that bind to these sites.

Binding site	Associated Residues	Allosteric Binder	PDB ID	Reference
Exo site	K14 G16 G17 N18 L63 E65 L70	4D9	3KFN	[107]
		Br7	N/A	[108]
Flap-top pocket	Y42 P44 M46 K55 R57 P79	1F1-N	4EJK	[109]
		AK-2097	4TVG	[109]
		Br6	2AZC	[110]
		DRV	3UCB	[111]
		ATV	3OXV	[112]
Eye Site	V32 I47 G48 G49 I50 I54 V56 G78 P79 T80 P81 I84	DHQB	N/A	[113]
		HIVE 9	5VJ3	N/A
		NIT	N/A	[114]
		5NI	N/A	[107]
Cantilever site	P1 Q2 I3 K7 T12 C67	HIVE 9	5W5W	N/A

## Data Availability

Not applicable.

## Supplementary Materials

The following supporting information can be downloaded at: https://www.mdpi.com/article/10.3390/v17081044/s1, molecular dynamics trajectory coordinates.
